# Violent crime exposure classification and adverse birth outcomes: a geographically-defined cohort study

**DOI:** 10.1186/1476-072X-5-22

**Published:** 2006-05-17

**Authors:** Lynne C Messer, Jay S Kaufman, Nancy Dole, Amy Herring, Barbara A Laraia

**Affiliations:** 1 US Environmental Protection Agency/National Human Environmental Effects Research Laboratory, Human Studies Division, MD 58A, Research Triangle Park, NC 27711, USA; 2 Department of Epidemiology, School of Public Health, CB # 7435, University of North Carolina, Chapel Hill, NC 27599-7435, USA; 3 Carolina Population Center, University of North Carolina, CB #8120, Chapel Hill, NC 27599-8120, USA; 4 Department of Biostatistics, School of Public Health, University of North Carolina, CB #7420, Chapel Hill, NC 27599-7420, USA

## Abstract

**Background:**

Area-level socioeconomic disparities have long been associated with adverse pregnancy outcomes. Crime is an important element of the neighborhood environment inadequately investigated in the reproductive and public health literature. When crime has been used in research, it has been variably defined, resulting in non-comparable associations across studies.

**Methods:**

Using geocoded linked birth record, crime and census data in multilevel models, this paper explored the relevance of four spatial violent crime exposures: two proximal violent crime categorizations (count of violent crime within a one-half mile radius of maternal residence and distance from maternal residence to nearest violent crime) and two area-level crime categorizations (count of violent crimes within a block group and block group rate of violent crimes) for adverse birth events among women in living in the city of Raleigh NC crime report area in 1999–2001. Models were adjusted for maternal age and education and area-level deprivation.

**Results:**

In black and white non-Hispanic race-stratified models, crime characterized as a proximal exposure was not able to distinguish between women experiencing adverse and women experiencing normal birth outcomes. Violent crime characterized as a neighborhood attribute was positively associated with preterm birth and low birth weight among non-Hispanic white and black women. No statistically significant interaction between area-deprivation and violent crime category was observed.

**Conclusion:**

Crime is variably categorized in the literature, with little rationale provided for crime type or categorization employed. This research represents the first time multiple crime categorizations have been directly compared in association with health outcomes. Finding an effect of area-level violent crime suggests crime may best be characterized as a neighborhood attribute with important implication for adverse birth outcomes.

## Background

The literature related to area-level effects, particularly socioeconomic disadvantage, on public health outcomes has grown substantially in recent years [1-18]. Research in perinatal health has demonstrated modest but consistent effects of neighborhood-level socioeconomic disparities in key pregnancy outcomes using census variables [4,5,19,20]. Low birth weights (LBW) have been associated with a variety of neighborhood level socioeconomic variables including poverty [8,11,12], unemployment [11], education and income [7,8,11] and median rent [8], as well as socio-demographic indices of economic disadvantage [15,17]. Area-level research has a long-standing tradition in the United Kingdom (U.K.) [21-24]. Established area-level indices such as the Townsend Material Deprivation Score and the Carstairs Deprivation Index have been widely utilized in the U.K., which have allowed for the comparison of deprivation effects across a variety of geographic regions. While the last decade has seen advancements in the field of neighborhood research in the United States (U.S.), including innovative data collection approaches [25-28] and increased access to relevant methodology such as multilevel modeling and geographic information systems (GIS) applications, the range of exposures used to represent relevant area-level effects has been largely limited to socio-demographic variables available from the census.

Census data, critical for identifying important associations between socioeconomic disadvantage and a variety of adverse health outcomes, are limited in their utility for public health research several reasons. First, census data are available only at decennial intervals in the US, whereas neighborhood condition and its subsequent effect can change within the span of a few years. Second, the exclusive use of census variables, which are produced by aggregating individual responses to census questions, implies that the important features of 'neighborhoods' can be captured by aggregating individual measures, ignoring the important role of other neighborhood features, such as the presence neighborhood of parks, the quality of area resources or the quantity of local disamenities such as land fills or strip clubs [29,30]. Third, census variables are limited to socio-demographic features. Economic and demographic features, while clearly important, are not the only neighborhood characteristics likely to affect health intermediates and outcomes. Fourth, census data are restricted to census geography, such as block groups and tracts, which may bear no resemblance to the salient features of 'neighborhoods' for most individuals. Therefore, while census variables continue to function as crude surrogates for neighborhood attributes, other aspects of the neighborhood must be assessed to elucidate more clearly the pathways through which neighborhoods might influence health.

Crime is a potentially important neighborhood characteristic inadequately examined in public health research despite documented relevance to birth outcomes, asthma and health behaviors, such as physical activity. Crime, and fear of crime, are both considered in the literature. Fear of crime is thought to contribute to an underlying mechanism explaining area differences in health [31-33] and has been directly associated with poor health outcomes in several studies [34-36], even after adjusting for health behaviors and a number of individual and household level socioeconomic factors [31]. Neighborhood-level crime is inversely associated with physical activity [37], especially among adolescent females [38], with important implications for youth overweight. Exposure to violence, including neighborhood violence, also predicts higher numbers of asthma symptom days and more nights of lost sleep for caretakers, after adjustment for socioeconomic status, housing deterioration and negative life events [39].

Four previous studies have reported an association between crime and poor pregnancy outcomes. In Santiago, Chile, Zapata and colleagues assigned ratings for sociopolitical violence and found exposure to violent environments associated with poor pregnancy outcomes, adjusting for individual-level risk factors [40]. In their research on impoverished women in Chicago (those living in census tracts with family median incomes <$10,000), Collins and David (1997) found more small-for-gestational-age and LBW deliveries among women living in high (16% LBW), compared with low crime rate neighborhoods (12% LBW). These relationships remained significant after controlling for individual risk factors [41]. Similarly, using the violent crime rate in Chicago, Morenoff (2003) found violent crime to be a robust neighborhood predictor of LBW after controlling for individual covariates [18]. Most recently, we found living in block groups in the highest and second highest quartiles of violent crime rate, compared with the lowest quartile of violent crime rate, was associated with increased odds of preterm birth among black non-Hispanic women (OR = 1.5, 95%CI: 0.9, 2.6, OR = 1.4, 95%CI: 1.0, 2.1, respectively) in adjusted models [42]. The relationship between neighborhood violence and birth outcomes appears suggestive and may help explain the disparity in birth outcomes between white and black women.

Crime exposure has been variably categorized in the literature and have included episodes of political violence [40,43,44], one-half mile violent crime density [38], violent crime incidents [45], and area crime rates [18,41,42,46]. Increased accessibility to geocoded data has enabled more sophisticated crime modeling techniques [47,48]. Crime is often reported as a rate or count for a specific geographic space, such as city, county or state. Geocoding, and the creation of distance-based measures to area resources or disamenities, has been an important innovation in health-related research [49-51]. Geocoding allows one to observe the spatial distribution of crime over multiple geographies to more clearly see the areas within a given locality (city, census tract) where crimes are most likely to be reported. It further allows the creation of different crime exposures, like distance to nearest crimes or count of crimes within a certain spatial area. The utility of different crime modeling techniques and the most relevant exposure for health outcomes has not been previously explored, despite probable implications for health.

Using the health example of adverse birth outcomes, this paper will explore the relevance of various violent crime categorizations by assessing their respective associations with two adverse birth outcomes, preterm birth and low birth weight. *A priori*, we hypothesized that proximal crime measures would be more predictive of adverse birth outcomes than area-level measures. Because no diagnostic criteria are available to formally compare the different exposure models, the predictive utility of each crime categorization, or that crime exposure categorization that proves most useful in differentiating women who experience an adverse birth outcome, will be considered most relevant. Previous published work on Wake County NC indicates black non-Hispanic and white non-Hispanic women live in different socioeconomic and demographic environments. Because of these known heterogeneities in neighborhood structures, which are associated with crime exposure, separate analyses were conducted for black non-Hispanic and white non-Hispanic women using multilevel logistic regression models.

## Methods

### Data sources

Exposure data from 1999–2001 City of Raleigh NC crime reports contain event locations, of which 99% were geocoded to latitude and longitude using Geographic Data Technology, Inc. (GDT) and assigned a crime category based on methods described in the literature [52]. These analyses are limited to violent crimes, including homicides, assaults, sexual assaults and kidnappings, of which 21,037 (22.5% of total crimes) occurred during the study period in Raleigh. Violent crime events were assigned to 2000 U.S. Census block groups to produce violent crime counts.

The birth outcome and maternal characteristics data are from 1999–2001 Wake County NC birth records (N = 30,481). Each maternal address identified on the birth certificate was geocoded to latitude and longitude and assigned to year 2000 U.S. census block groups. Of the 98.6% of birth records with complete addresses sent to GDT for geocoding, 93.2% achieved an exact census tract match using GDT's methods. The North Carolina birth records contain birth outcome, personal characteristics and health behavior information on each woman.

The third data source is year 2000 U.S. census data, from which the deprivation score, a neighborhood control variable, was derived. The deprivation score is a single summary representing four socioeconomic domains including poverty, housing, employment and education [53].

### Neighborhood definition

Neighborhood is a term loosely used to refer to a person's immediate residential environment, which is hypothesized to have both material and social characteristics related to health [54,55]. Census block groups were used to approximate the neighborhood environment. This level of aggregation is large enough to contain women who delivered during the study years, but small enough to approximate the immediate physical neighborhood for study subjects. Previous research has advocated using the smallest possible level of aggregation due to the considerable crime variability within larger ecological units [25,56].

### Violent crime exposures

The study was limited to women who resided in the Raleigh crime reporting area and were therefore subject to influence by reported area-level crimes (N = 13,960). Wake County comprises 263 block groups of which 114 represent the Raleigh crime report area. Crime exposure was conceptualized in two distinct ways: as an attribute of the neighborhood and as an attribute of the individual.

#### Neighborhood-level crime variables

At the neighborhood level, block group measures include a) block group violent crime count and b) block group violent crime rate per thousand population ([block group violent crime count / block group population] × 1000). Neighborhood crime tertiles were created based on block group violent crime distributions with 38 block groups per tertile, then merged with maternal data, resulting in common neighborhood categories for black non-Hispanic (hereafter referred to as "black") and white non-Hispanic (hereafter referred to as "white") women.

#### Proximal crime variables

Two variables conceptualize violent exposure to crime as an individual-level attribute, subject to the geographic distribution of the women in the sample. Proximal violent crime exposure was defined as: a) the count of violent crimes within a one-half mile radius of each woman's address and b) the distance in feet from maternal residence to the nearest violent crime. Previous work [42] and preliminary analyses indicated white and black women were exposed to different quantities of violent crime. Creating proximal crime cutpoints based on the combined distribution of black and white women resulted in the majority of white women falling in the lowest crime categories and the majority of black women falling in the highest crime categories, with virtually no representation of the other racial group in the extremes of the violent crime distribution. Using race-specific cutpoints allowed for a more equal distribution of women of both races across the continuum of individually defined crime. For these reasons, race-specific tertiles were created for the two variables that defined violent crime as an individual attribute. Furthermore, the geographic area represented by these proximal crime exposures differed substantially from the block group-level crime variables. Raleigh NC block groups are quite large, with a mean size of 1.26 square miles (range: 0.10, 15.64). These proximal crime variables represented a much smaller unit of geographic space around the study women.

### Outcome definition

This research explored the association between crime and two adverse birth outcomes, preterm birth and low birth weight (LBW). Preterm birth is defined as birth at gestational age <37 weeks and weighing less than 3,888 grams [57]. Clinically estimated gestational age was obtained from the birth record. Comparisons between clinically estimated gestational age and last menstrual period calculated gestational age found that for Wake County births, the clinical estimate better approximated the expected preterm, term and post-term proportions that were obtained from a clinical sample with ultrasound dated gestational age. For this reason, the clinical estimate of gestational age, combined with the weight restriction for preterm births, was used to calculate preterm birth. Less than one percent of the records were missing gestational age information. Low birth weight is defined as birth at less than 2500 grams. The vital records were missing no birth weight data. The birth cohort for this analysis was limited to singleton births.

### Covariates

Individual covariates considered include maternal age, education and marital status. These individual-level variables are established risk factors for preterm birth and possible confounders to the neighborhood crime-preterm birth relationship. The neighborhood-level covariate considered for this analysis was neighborhood deprivation and was controlled using a neighborhood deprivation score. The deprivation score is the weighted sum of nine standardized census variables including block group percents of households below 1999 poverty level, female headed households with dependent children, earning < $30,000 per year, on public assistance, with no car, selected owner and renter costs in excess of 50% of income, unemployment, individuals over age 25 with less than a high school education and median household value. The deprivation index has a median value of -0.4, a mean of 0.3 and standard deviation of 2.3 and a range of -2.3 to +12.5. The low end of the deprivation score indicates lack of deprivation (i.e., affluence) whereas a high end of the range suggests a large amount of deprivation. Continuous and categorical forms of the covariates were considered and the categorical forms used in the models. Location of police substation, while associated with crime reporting, is not associated with birth outcomes [42] and was not included as a neighborhood covariate in these analyses.

### Data analysis

Distributions and prevalence ratios of each exposure variable and individual and neighborhood-level covariates were examined. Analyses of variance (ANOVA) tested if mean neighborhood-level characteristics differed by block-group violent crime rate tertile. Race-specific multilevel logistic regression analyses were conducted to explore the contribution of the neighborhood environment (second level variables) over that of the individual-level predictors and to account for any clustering of the birth outcomes. The authors estimated random effects logistic models with a fixed slope value for each predictor variable but with block group-specific intercepts, adjusting the models for individual and neighborhood covariates. Adjustment for confounders was made when the crude odds ratio differed from the adjusted odds ratio by 10% or more [58]. Terms for the interaction between neighborhood deprivation and crime were introduced to assess if these terms improved the model fit. Analyses were conducted in Stata 8.2.

## Results

Of the 11,256 non-Hispanic women delivering singleton live births in Raleigh NC during the study years, 471 (6.7% of 7036) white and 539 (12.8% of 4220) black women delivered preterm and 308 (4.4%) white and 485 (11.5%) black women delivered a LBW infant (Table 1). White women in this sample were generally older with more years of education than black women. Black women were exposed on average to four times as many violent crimes within a half-mile radius: mean number = 106.9 (standard deviation = 139.1) and lived closer to the nearest violent crime: mean = 377.8 (745.9) feet compared with white women: who were exposed to, on average, 24.7 (43.2) crimes and lived on average 1173 (1414) feet from the nearest violent crime (Table 2).

**Table 1:** Individual-level characteristics of study sample, Raleigh crime report area, 1999–2001*

		**NH White N (%)**	**NH Black N (%)**	**% PTB**	**% LBW**	PR** (95% CI)
**Preterm birth**		471 (6.7)	539 (12.8)	8.7	70.6	1.91 (1.69, 2.15)
**Low birth weight**		308 (4.4)	485 (11.5)	70.6	6.9	2.49 (2.17, 2.87)
(column percent) **Individual-level maternal characteristics**						
**Marital status**						
	**Married**	6611 (89.7)	1815 (41.5)	7.3	5.2	0.46 (0.44, 0.48)
	**Not married**	761 (10.3)	2562 (58.5)	11.8	10.6	5.70 (5.30, 6.12)
**Maternal age**						
	**< 20 years**	210 (2.9)	555 (12.7)	9.7	9.9	4.45 (3.81, 5.19)
	**20–24 years**	772 (10.5)	1344 (30.7)	9.4	8.2	3.11 (2.87, 3.38)
	**25–29 years**	2000 (27.1)	1155 (27.4)	8.7	6.1	0.97 (0.91, 1.03)
	**30–34 years**	2798 (38.0)	823 (18.8)	7.5	5.8	0.50 (0.46, 0.53)
	**35+ years**	1592 (21.6)	500 (11.4)	9.3	6.8	0.53 (0.48, 0.58)
**Maternal education**						
	**< 12 years**	309 (4.2)	794 (18.2)	10.3	8.6	4.33 (3.82, 4.91)
	**12 years**	936 (12.7)	1350 (30.9)	10.6	9.2	2.43 (2.26, 2.62)
	**> 12 years**	6110 (83.1)	2219 (50.9)	7.6	5.6	0.61 (0.59, 0.63)

* Distribution, percent preterm birth, percent low birth weight and prevalence ratio [PR] (95% confidence intervals [95% CI]) for individual-level attributes of women living in Raleigh crime report area, 1999–2001

**PR = prevalence in blacks/prevalence in whites

**Table 2:** Proximal violent crime characteristics of study sample, Raleigh crime report area, 1999–2001*

(column percent)	**NH White N (%)**	**NH Black N (%)**	**% PTB**	**% LBW**	PR** (95% CI)
**Count of violent crimes within one-half mile of all maternal address**					
**Low (0 – 12)**	3049 (52.6)	735 (19.0)	7.1	5.3	0.36 (0.34, 0.39)
**Medium (13 – 49)**	2014 (34.7)	1098 (28.3)	9.0	7.3	0.82 (0.77, 0.87)
**High (50 – 633)**	736 (12.7)	2043 (52.7)	10.2	8.8	4.15 (3.86, 4.47)
**Distance in feet from maternal residence to nearest violent crime**					
**Close (0 – 89)**	829 (12.7)	2120 (51.9)	10.2	8.9	4.09 (3.82, 4.39)
**Med (89 – 771)**	2279 (34.9)	1387 (34.0)	8.7	7.0	0.97 (0.92, 1.03)
**Far (772 – 15,616)**	3430 (52.5)	577 (14.1)	6.8	4.8	0.27 (0.25, 0.29)

* Distribution, percent preterm, percent low birth weight and prevalence ratios [PR] (95% confidence intervals [95% CI]) for proximal crime variables for women living in Raleigh crime report area, 1999–2001

**PR = prevalence in blacks/prevalence in whites

Levels of neighborhood deprivation also differed by race (Table 3), with 54.2% of white women living in the block groups in the lowest tertile of deprivation (most affluent) compared with a roughly equal proportion of black women, 52.1%, living in the most deprived neighborhoods, or those block groups in the highest tertile of deprivation. Consistent with this pattern of exposure to neighborhood deprivation, black women lived in block groups with more violent crimes (68.6 [53.1]) and a higher violent crime rate (31.4 [37.2]) than white women (27.4 [36.1] and 8.5 [16.5], respectively) in this study.

**Table 3:** Area-level deprivation and crime characteristics of study sample, Raleigh crime report area, 1999–2001*

(column percent)	**NH White N (%)**	**NH Black N (%)**	**% PTB**	**% LBW**	PR** (95% CI)
**Neighborhood-level deprivation**					
**Neighborhood deprivation**					
**Low ([-2.8]–[-0.7])**	3922 (54.2)	659 (15.1)	6.8	4.8	0.28 (0.26, 0.30)
**Med ([-0.6]–0.8)**	2667 (36.2)	1436 (32.8)	8.7	6.8	0.91 (0.86, 0.96)
**High (0.82 – 12.5)**	713 (9.7)	2282 (52.1)	11.0	9.7	5.39 (5.00, 5.81)
**Neighborhood-level violent crime**					
**Neighborhood violent crime count**					
**Low (0 – 12)**	3176 (43.1)	583 (13.3)	6.8	4.7	0.31 (0.29, 0.33)
**Medium (13 – 50)**	3019 (41.0)	1348 (30.8)	8.6	6.7	0.75 (0.71, 0.79)
**High (52 – 378)**	1177 (16.0)	2446 (55.9)	10.4	8.8	3.50 (3.30, 3.71)
**Neighborhood violent crime rate**					
**Low (0 – 6)**	4553 (61.8)	920 (21.0)	7.0	4.9	0.34 (0.32, 0.36)
**Medium (6 – 16)**	2138 (29.0)	1404 (32.1)	9.1	7.2	1.11 (1.05, 1.17)
**High (17 – 205)**	681 (9.2)	2053 (46.9)	11.0	9.6	5.07 (4.70, 5.49)

* Distribution, percent preterm, percent low birth weight and prevalence ratios [PR] (95% confidence intervals [95% CI]) for neighborhood-level deprivation and violent crime variables for women living in Raleigh crime report area, 1999–2001

**PR = prevalence in blacks/prevalence in whites

Neighborhood-level features were associated with block group crime tertiles in Raleigh NC (Table 4). Block groups with low rates of violent crime (tertile 1) generally had less poverty (6.1%) and unemployment (2.3%) and fewer households with the following characteristics: female headed with dependent children (7.1%), earning < $30,000/year (19.5%), on public assistance (1.2%), with no car (1.4%) and with low education (6.4%) compared with block groups characterized by high rates of violent crime (tertile 3). Block group level median home value decreased as block groups experienced more violent crimes from approximately $22,000 to $12,000. Interestingly, one neighborhood feature that did not differ across crime tertiles was high housing costs; housing costs appear generally high across Raleigh block groups, regardless of crime rate. The correlation between the neighborhood deprivation index and the continuous violent crime rate was 0.55.

**Table 4:** Association between area-level characteristics and tertiles of block group crime rate 1999–2001*

Mean percent (standard deviation)	**Tertile 1: (0.0 – 0.005)**	**Tertile 2: (0.006 – 0.019)**	**Tertile 3: (0.022 – 0.21)**	**F-test P-value**
**Black Non-Hispanic**	9.7 (11.5)	24.3 (21.5)	52.0 (32.2)	P < 0.001
Poverty**	6.1 (5.4)	11.7 (9.9)	22.1 (14.8)	P < 0.001
Female headed HH***	7.1 (4.9)	12.2 (7.9)	21.9 (17.5)	P < 0.001
**HH income <$30,000**	19.5 (10.3)	30.1 (15.3)	47.4 (18.1)	P < 0.001
**Public assistance**	1.2 (1.4)	1.5 (2.8)	5.3 (5.5)	P < 0.001
**No car**	1.4 (1.4)	1.9 (2.4)	8.0 (12.4)	P < 0.001
High housing costs****	23.0 (12.7)	24.5 (10.2)	27.0 (10.1)	P = 0.29
**Unemployment**	2.3 (2.3)	4.2 (5.2)	9.8 (11.7)	P < 0.001
Low education*****	6.4 (7.7)	11.5 (9.1)	25.5 (14.6)	P < 0.001
Median home value******	22.0 (88.3)	17.4 (11.1)	12.0 (60.7)	P < 0.001

* Percent of block group characteristics and F-statistic P-value comparing mean values for block group crime rate tertiles in Raleigh crime report area, 1999–2001.

** Percent households reporting living under the 1999 federal poverty line

*** Percent households headed by females with dependent children

**** Specified owner or renter costs in excess of 50% of income

***** Less than 12 years of education among adults 25 years or older

****** Median household value(× $10000.00)

White women's odds of adverse birth outcomes appear modestly associated with violent crime in this sample. The proximal exposure categorizations of violent crime, such as the count of violent crimes within a half-mile and distance to nearest violent crimes (Table 5), do not distinguish women at increased odds of delivering preterm; the odds ratios in adjusted models remain close to the null. Exposure to high counts of violent crimes within a half-mile radius of maternal residence suggests a small association with both preterm birth and LBW in unadjusted models, but following adjustment, the association is attenuated. White women living in neighborhoods with high, compared with low rates of violent crime (Table 6) appear to be at increased odds of preterm birth (OR= 1.4; 95% CI: 1.1, 1.9) and LBW (OR = 1.7; 95% CI: 1.2, 2.4). The relationship between living in a block group with a high violent crime rate (between 17 and 205 crimes per 1000 population) and adverse birth outcomes is attenuated following adjustment for individual and neighborhood covariates, though still suggestive of increased odds for LBW (OR = 1.5; 95% CI: 1.0, 2.3) but less so for preterm birth (OR = 1.3; 95% CI: 0.9, 1.9).

**Table 5:** Associations between proximal crime exposures and adverse birth outcomes among non-Hispanic white women*

	**Preterm birth (<37 weeks gestation & <3888 g)**				**Low birth weight (<2500 g)**			
	**Not adjusted**	**Individual covariates**	**Area-level covariates**	**Fully adjusted**	**Not adjusted**	**Individual covariates**	**Area-level covariates**	**Fully adjusted**

**Count of violent crimes within 1/2 mile of maternal address (White non-Hispanic cutpoints used in model)**								
**Low (0 – 6)**	Referent	Referent	Referent	Referent	Referent	Referent	Referent	Referent
**Medium (7 – 21)**	1.0 (0.8, 1.4)	1.0 (0.8, 1.3)	1.0 (0.8, 1.3)	1.0 (0.8, 1.3)	1.1 (0.8, 1.5)	1.0 (0.7, 1.4)	1.0 (0.7, 1.4)	1.0 (0.7, 1.4)
**High (22 – 562)**	1.2 (1.0, 1.6)	1.1 (0.9, 1.5)	1.1 (0.8, 1.6)	1.1 (0.8, 1.5)	1.2 (0.9, 1.7)	1.1 (0.8, 1.5)	1.1 (0.8, 1.7)	1.0 (0.7, 1.5)
**Distance (in feet) from maternal address to nearest violent crime (White non-Hispanic cutpoints used in model)**								
**Close (0–512)**	Referent	Referent	Referent	Referent	Referent	Referent	Referent	Referent
**Medium (513–1239)**	1.0 (0.8, 1.3)	1.1 (0.8, 1.4)	1.1 (0.8, 1.4)	1.1 (0.9, 1.5)	0.8 (0.6, 1.1)	0.9 (0.7, 1.2)	0.8 (0.6, 1.1)	0.9 (0.7, 1.2)
**Far (<15,617)**	1.0 (0.8, 1.3)	1.1 (0.9, 1.4)	1.1 (0.8, 1.4)	1.2 (0.9, 1.5)	0.8 (0.6, 1.1)	0.9 (0.7, 1.3)	0.9 (0.6, 1.2)	1.0 (0.7, 1.3)

* Unadjusted, individual (maternal age, education), neighborhood (area-level deprivation), and fully-adjusted (individual and area-level covarites) odds ratios [OR] (95% Confidence Intervals [95% CI]) of preterm birth and low birth weight for 1/2 mile violent crime count and distance to nearest violent crime among non-Hispanic white women living in Raleigh NC.

**Table 6:** Associations between area-level violent crime and adverse birth outcomes among non-Hispanic white women*

	**Preterm birth (<37 weeks gestation & <3888 g)**				**Low birth weight (<2500 g)**			
	**Not adjusted**	**Individual covariates**	**Area-level covariates**	**Fully adjusted**	**Not adjusted**	**Individual covariates**	**Area-level covariates**	**Fully adjusted**

**Block group violent crime count**								
**Low (0 – 12)**	Referent	Referent	Referent	Referent	Referent	Referent	Referent	Referent
**Med (13 – 50)**	1.0 (0.8, 1.2)	1.0 (0.8, 1.2)	1.0 (0.8, 1.2)	0.9 (0.8, 1.2)	1.0 (0.8, 1.3)	1.0 (0.8, 1.3)	1.0 (0.8, 1.3)	1.0 (0.8, 1.3)
**High (52 – 378)**	1.2 (0.9, 1.6)	1.1 (0.8, 1.4)	1.1 (0.8, 1.5)	1.1 (0.8, 1.5)	1.2 (0.9, 1.6)	1.0 (0.8, 1.5)	1.0 (0.7, 1.5)	1.0 (0.7, 1.4)
Block group violent crime rate (count/population * 1000)								
**Low (0 – 5.7)**	Referent	Referent	Referent	Referent	Referent	Referent	Referent	Referent
**Medium (6.1–16.3)**	1.0 (0.8, 1.2)	0.9 (0.7, 1.2)	1.0 (0.8, 1.2)	1.0 (0.7, 1.2)	1.0 (0.8, 1.3)	1.0 (0.7, 1.3)	1.0 (0.7, 1.4)	1.0 (0.7, 1.3)
**High (<204.7)**	1.4 (1.1, 1.9)	1.3 (1.0, 1.8)	1.3 (0.9, 1.9)	1.3 (0.9, 1.9)	1.7 (1.2, 2.4)	1.5 (1.1, 2.2)	1.6 (1.0, 2.4)	1.5 (1.0, 2.3)

* Unadjusted, individual (maternal age, education), neighborhood (area-level deprivation), and fully-adjusted (individual and area-level covarites) odds ratios [OR] (95% Confidence Intervals [95% CI]) of preterm birth and low birth weight for area-level violent crime count and violent crime rate among non-Hispanic white women living in Raleigh NC.

Similar to the results observed for white women, crime categorized as a proximal exposure shows little association with adverse birth outcomes for black women. In unadjusted models (Table 7), living far from the nearest violent crime suggests protection against LBW (OR = 0.8, 95% CI: 0.6, 1.0), which is the direction of the relationship one might anticipate, but this association is attenuated following adjustment for individual covariates (OR = 0.9, 95% CI: 0.7, 1.1). Violent crime defined as a neighborhood attribute, however, appears to be modestly associated with adverse birth outcomes among black women. In unadjusted models (Table 8), living in a block group with medium and high counts of violent crime confers 80% and 60% increased odds of preterm birth (OR = 1.8, 95% CI: 1.3, 2.5, and OR = 1.6, 95% CI: 1.2, 2.3, respectively). Following adjustment, odds of preterm birth are reduced for the highest tertile (OR = 1.2, 95% CI: 0.9, 1.8), but remain associated for the middle tertile (OR = 1.7, 95% CI: 1.2, 2.4). A similar pattern is apparent for LBW; medium levels of violent crime count are associated with LBW after adjustment for individual covariates (OR = 1.6, 95% CI: 1.1, 2.4) while high levels of violent crime count appear to confer reduced odds of low birth following adjustment (OR = 1.2, 95% CI: 0.8, 1.7). In the models employing violent crime rates, odds for adverse birth outcomes show a similar pattern. Unadjusted results suggest living in block groups characterized by medium (OR = 1.6; 95% CI: 1.2, 2.0) or high (OR = 1.5; 95% CI: 1.1, 1.9) rates of violent crime are associated with increased odds of preterm birth, compared with living in low violent crime rate block groups. Following adjustment for covariates, the associations are attenuated (OR = 1.3; 95% CI: 1.0, 1.8 and OR = 1.1; 95% CI: 0.8, 1.6) but remain modestly associated for medium violent crime rate block groups. Similarly, the odds of LBW are associated with medium (OR = 1.3; 95% CI: 1.0, 1.8) and high rates of violent crime (OR = 1.5; 95% CI: 1.2, 2.0) in unadjusted models, but these relationships are reduced following adjustment (OR = 1.2, 95% CI: 0.9, 1.7 and OR = 1.2, 95% CI: 0.8, 1.7, respectively).

**Table 7:** Associations between proximal violent crime exposures and adverse birth outcomes among non-Hispanic black women*

	**Preterm birth (<37 weeks gestation & <3888 g)**				**Low birth weight (<2500 g)**			
	**Not adjusted**	**Individual covariates**	**Area-level covariates**	**Fully adjusted**	**Not adjusted**	**Individual covariates**	**Area-level covariates**	**Fully adjusted**

**Count of violent crimes within 1/2 mile of maternal address (Black non-Hispanic cutpoints used in this model)**								
**Low (0 – 29)**	Referent	Referent	Referent	Referent	Referent	Referent	Referent	Referent
**Medium(30 – 81)**	1.0 (0.8, 1.3)	0.9 (0.7, 1.2)	0.9 (0.7, 1.2)	0.9 (0.7, 1.2)	1.0 (0.8, 1.3)	0.9 (0.7, 1.2)	0.9 (0.7, 1.2)	0.9 (0.6, 1.1)
**High (82 – 633)**	1.1 (0.9, 1.4)	1.0 (0.8, 1.3)	1.0 (0.7, 1.4)	0.9 (0.7, 1.3)	1.3 (1.0, 1.6)	1.1 (0.8, 1.4)	1.1 (0.8, 1.5)	1.0 (0.7, 1.3)
**Distance (in feet) from maternal address to nearest violent crime (Black non-Hispanic cutpoints used in model)**								
**Close (0 – 21)**	Referent	Referent	Referent	Referent	Referent	Referent	Referent	Referent
**Medium (22 – 287)**	1.1 (0.9, 1.4)	1.1 (0.9, 1.4)	1.1 (0.9,1.4)	1.1 (0.9, 1.4)	1.0 (0.8, 1.3)	1.1 (0.8, 1.4)	1.0 (0.8, 1.3)	1.1 (0.8, 1.4)
**Far (<12857)**	0.8 (0.7, 1.1)	0.9 (0.7, 1.2)	0.9 (0.7, 1.1)	0.9 (0.7,1.2)	0.8 (0.6, 1.0)	0.8 (0.6, 1.1)	0.8 (0.6, 1.0)	0.9 (0.7, 1.1)

** Unadjusted, individual (maternal age, education), neighborhood (area-level deprivation), and fully-adjusted (individual and area-level covarites) odds ratios [OR] (95% Confidence Intervals [95% CI]) of preterm birth and low birth weight for 1/2 mile violent crime count and distance to nearest violent crime among non-Hispanic black women living in Raleigh NC.

**Table 8:** Associations between area-level violent crime and adverse birth outcomes among non-Hispanic black women*

	**Preterm birth (<37 weeks gestation & <3888 g)**				**Low birth weight (<2500 g)**			
	**Not adjusted**	**Individual covariates**	**Area-level covariates**	**Fully adjusted**	**Not adjusted**	**Individual covariates**	**Area-level covariates**	**Fully adjusted**

**Block group violent crime count**								
**Low (0 – 12)**	Referent	Referent	Referent	Referent	Referent	Referent	Referent	Referent
**Med (13 – 50)**	1.8 (1.3, 2.5)	1.8 (1.3, 2.5)	1.7 (1.2, 2.4)	1.7 (1.2, 2.4)	1.6 (1.1, 2.3)	1.7 (1.2, 2.4)	1.6 (1.1, 2.3)	1.6 (1.1, 2.4)
**High (52 – 378)**	1.6 (1.2, 2.3)	1.5 (1.1, 2.1)	1.3 (0.9, 1.9)	1.2 (0.9, 1.8)	1.6 (1.2, 2.2)	1.4 (1.0, 2.0)	1.3 (0.9, 1.9)	1.2 (0.8, 1.7)
Block group violent crime rate (count/population * 1000)								
**Low (0 – 5.7)**	Referent	Referent	Referent	Referent	Referent	Referent	Referent	Referent
**Medium (6.1–16.3)**	1.6 (1.2, 2.0)	1.5 (1.2, 2.0)	1.4 (1.0, 1.8)	1.3 (1.0, 1.8)	1.3 (1.0, 1.8)	1.3 (1.0, 1.8)	1.2 (0.9, 1.7)	1.2 (0.9, 1.7)
**High (<204.7)**	1.5 (1.1, 1.9)	1.3 (1.0, 1.8)	1.2 (0.9, 1.7)	1.1 (0.8, 1.6)	1.5 (1.2, 2.0)	1.3 (1.0, 1.8)	1.3 (0.9, 1.8)	1.2 (0.8, 1.7)

* Unadjusted, individual (maternal age, education), neighborhood (area-level deprivation), and fully-adjusted (individual and area-level covarites) odds ratios [OR] (95% Confidence Intervals [95% CI]) of preterm birth and low birth weight for area-level violent crime count and violent crime rate among non-Hispanic black women living in Raleigh NC.

To examine if the interaction between neighborhood deprivation and crime influenced birth outcomes, we developed 8 models interacting the dichotomized deprivation index with each category of violent crime. Based on likelihood ratio tests, none of the interaction models was an improvement over the main effect models (p-values ranging from 0.3 – 0.8). In each interaction model, the adjusted odds ratios were consistent with the main effect models.

## Discussion

The measurement of neighborhood effects on health has generally been imprecise, in part due to conceptual and methodological limitations [59]. Researchers often have to make use of administrative or other types of data not explicitly collected for research on health outcomes that offer few options for variable creation and exposure categorization. Using geocoded crime report data, this research sought to contribute to the neighborhood effects literature by testing various spatial and area-level violent crime exposure categorizations and assessing their association with adverse birth outcomes.

Crime was grouped into two main categories: attributes of individuals, or proximal measures, and attributes of neighborhoods. Crimes characterized as proximal, including the count of violent crimes within a half mile of maternal address and distance from maternal address to nearest violent crime were less predictive than those estimating the neighborhood environment in associations with adverse birth outcomes.

Among white women, the rate of neighborhood violent crime showed the strongest association with low birth weight, and a modest association with preterm birth. Several possible explanations exist for this modest effect. First, it is possible that violent crime has little effect on birth outcomes. This potential explanation is somewhat refuted by the violent crime effect observed in other studies. And while possible, this explanation seems unlikely given the research showing that women, and particularly white women, are more fearful of crime than others, despite their decreased risk of victimization compared with other racial and gender groups [60]. The second explanation for the modest violent crime effect is that the white women in this area are minimally exposed. The research reported here supports this explanation. Well over half the white women in this study had low counts of violent crimes within a one-half mile, lived in the farthest tertile from the nearest violent crime, and in block groups with the lowest counts and rate of violent crimes. A related explanation is that the relatively crime free and affluent neighborhoods in which the majority of white women in Raleigh reside offer some protection against, or buffer against, the possibly harmful effects of crime exposure. This explanation is at least partially supported by the study findings, as well. Observing an effect of neighborhood crime rate for white women living in block groups characterized by the highest tertile of violent crime is suggestive and supports the need for further investigation of crime as a neighborhood phenomenon.

Finding the strongest violent crime effect among black women living in the middle, compared with the lowest tertile of violent crime count was unanticipated. One possible explanation for this finding lies in the fact these middle tertile block groups are more racially heterogeneous than the neighborhoods characterized by the upper tertiles of violent crime and previous research has found racial homogeneity to be of some health benefit [6,61]. Neighborhoods in the middle tertile of violent crime house more white women; black women living in these more heterogeneous neighborhoods may be exposed to other, unmeasured stressors that women at the extremes do not face. One candidate stressor is interpersonal or institutional racism, which may affect birth outcomes. Additionally, black women living in the highest tertile of violent crime are simultaneously exposed to multiple economic stressors. While one might anticipate economic deprivation would lead to increased odds of adverse birth outcomes, as multiple studies have found, these women may have psychologically and physiologically accommodated this increased level of environmental stress and become relatively inured to its effects. In the absence of better individual-level exposure assessment and information regarding women's coping with and perceptions of crime, it is difficult to know what unmeasured individual or neighborhood confounders may influence the association between crime and birth outcomes and put the black women in this middle crime tertile at a relative disadvantage.

Finding an effect of neighborhood-level violent crime is consistent with previous research. In their research on impoverished women in Chicago (those living in census tracts with family median incomes <$10,000), Collins and David (1997) found more small-for-gestational-age and LBW deliveries among women living in high, compared with low crime rate neighborhoods. Similarly, using the violent crime rate in Chicago, Morenoff found violent crime to be a robust neighborhood predictor of LBW after controlling for individual covariates [18]. The research reported here confirms previous low birth weight studies and expands birth outcomes consideration to the etiologically significant outcome of preterm birth. This work builds on earlier findings, which focus largely on violent crime rates, by categorizing crime exposure in multiple ways. It was interesting to note that for white women, relative quantities of violent crime, or the violent crime rate, appeared associated with adverse birth outcomes whereas for black women, absolute violent crime (the violent crime count) appeared more influential.

Finding no effect on birth outcomes for living in close proximity to violent crime was unexpected. We anticipated living farther from violent crime would be protective against preterm birth while living within a half mile of a large number of violent crime episodes would increase a woman's risk of preterm birth. These expectations were not borne out. It is possible that the measurement error associated with geocoded crime events and maternal addresses is sufficiently large to preclude capturing salient distances or densities of violent crime or that systematic crime reporting errors may result in crime misclassification. Additionally, crime report data does not represent actual crime experiences and in the absence of daily diaries or other individual data collection techniques, it is impossible to assess actual crime exposure.

The mechanisms through which crime may influence health, and birth outcomes in particular, are uncertain. Exposure to violent environments has been associated with mental health in children [62] and sleep disturbances, nightmares and other anxiety manifestations among adults [63]. Another possible mechanism by which crime can influence health is through chronic stress. Research in this area is new, but some work has found violence exposure to be an important constituent of chronic environmental stress, suggested to play a role in developing essential hypertension through elevated sympathetic nervous system activity [45]. Other work finds evidence for an association between maternal stress or stressful life events and adverse pregnancy outcomes [64-68], including PTB [67-69], while other research does not [70,71]. Crime environments may influence health outcomes through behavior. Crime has been previously associated with health risk behaviors in young men [72] and women [73]. Health behaviors, especially those used to reduce stress such as cigarette smoking and alcohol consumption, can be particularly harmful during pregnancy.

This research is limited in several ways. The choice of control variables was limited to those reliably collected on the birth record. Other covariates, including tobacco and alcohol use, may partially explain the study results, but because of data quality issues were not explored in these analyses. Further, recognizing the presence of a crime event may differentially impact pregnancy health depending on perceptions of overall neighborhood quality, threat, pre-existing anxieties and a host of other psychosocial factors not assessed in this research. This study is further limited by its reliance on administratively defined boundaries to approximate the 'neighborhood'. The census block group is a relatively small unit of aggregation, but may bear no resemblance to the salient neighborhood-level exposure. Further, each woman's definition of neighborhood may differ, adding another level of complexity to determining relevant exposure types and levels. Despite the potential misattribution of "neighborhood" influence to an administrative unit, other authors have found using the census block group as the unit of analysis useful in studies of birth outcomes [17]. The study relied on birth certificate data for all individual-level data; the quality of birth record data is variable. Among North Carolina birth certificates, research indicates reporting is very accurate for birth weight and fair to good for most other variables, but poor for medical history and alcohol use [74]. The greatest concern with using birth record data in this research involves the construction of gestational age, from which one outcome measure is obtained. This limitation has been addressed through use of the clinical gestational age estimate, as discussed in the methods section.

One issue that frequently perplexes epidemiologic research is that of the modifiable areal unit problem [MAUP]. The MAUP arises from the imposition of artificial units of spatial reporting on continuous geographical phenomenon resulting in the generation of artificial spatial patterns [75]. The MAUP describes two effects that influence statistical and epidemiological results: scale and aggregation effects. The scale effect produces different statistical results by altering the denominator within the same dataset [76]. The aggregation or zoning effect arises from variability in the way units can be grouped at a given scale [76]. By assessing both census tract (data not shown) and census block group data, and finding similar effects at both levels of aggregation, this research can be considered robust to the scale effects of the MAUP. While this research did not address zoning effects directly, its use of different spatial categorizations of crime was an attempt to forward the field of area-level effects on health outcomes.

This research represents an important step in refining neighborhood exposures for health outcomes. The use of geocoded data allowed for multiple violent crime categorization forms. To our knowledge, this paper is the first to employ multiple categorizations of crime exposure and assess the relationship of each to a health outcome. Additionally, this paper considered two adverse birth outcomes, thereby providing a broader array of information. The study benefited from a large number of women, births and crime events; these numbers enabled the investigators to observe modest effects on relatively rare outcomes.

## Conclusion

Living in close proximity to crime, estimated by the count of violent crimes within a half-mile radius of residence and the distance from residence to nearest violent crime, was not associated with adverse birth outcomes in this research. Area-level crime, whether measured as the count of violent crimes within a given block group or the block group rate of violent crime, was more useful for differentiating areas where women would and would not be at increased odds of an adverse birth outcome, even after adjusting for neighborhood-level deprivation. This is the first work to consider multiple categorizations of crime exposure in association with health outcomes. The results from this paper suggest that, at least for birth outcomes, crime appears best considered as part of the general neighborhood environment, rather than as a proximal exposure.

Preterm birth and LBW are important public health outcomes, but pregnancy is a resilient time for many women. Most pregnancies can endure a variety of 'insults' and still result in healthy, normal weight, term infants. Other health outcomes may prove more sensitive to the 'individual exposures' approach to crime characterization and this possibility should be considered in further research.

## Competing interests

The author(s) declare that they have no competing interests.

## Authors' contributions

LCM conceived of and drafted the manuscript and conducted the study analyses. JSK co-conceived of the manuscript and participated in the study design and analysis plan. NDR participated in the study conception, design and coordination. AH contributed to the statistical analyses and modifications of the manuscript. BAL helped conceived of the study, and participated in its design and coordination. All authors read and approved the final manuscript.
